# Research on Classification Model of *Panax notoginseng* Taproots Based on Machine Vision Feature Fusion

**DOI:** 10.3390/s21237945

**Published:** 2021-11-28

**Authors:** Yinlong Zhu, Fujie Zhang, Lixia Li, Yuhao Lin, Zhongxiong Zhang, Lei Shi, Huan Tao, Tao Qin

**Affiliations:** 1 Faculty of Modern Agricultural Engineering, Kunming University of Science and Technology, Kunming 650500, China; 20192114019@stu.kust.edu.cn (Y.Z.); 20030031@kust.edu.cn (F.Z.); 20202114016@stu.kust.edu.cn (Y.L.); shilei04@stu.kust.edu.cn (L.S.); 20192214014@stu.kust.edu.cn (H.T.); 20192114001@stu.kust.edu.cn (T.Q.); 2 Engineering Research Center of Mechanization of Chinese Medicinal Materials of Yunnan Province, Kunming 650500, China; 3 College of Mechanical and Electronic Engineering, Northwest A&F University, Xianyang 712100, China; zzx9519@nwsuaf.edu.cn

**Keywords:** machine vision, machine learning, *Panax notoginseng* taproot, feature fusion, image processing, hierarchical model

## Abstract

The existing classification methods for *Panax notoginseng* taproots suffer from low accuracy, low efficiency, and poor stability. In this study, a classification model based on image feature fusion is established for *Panax notoginseng* taproots. The images of *Panax notoginseng* taproots collected in the experiment are preprocessed by Gaussian filtering, binarization, and morphological methods. Then, a total of 40 features are extracted, including size and shape features, HSV and RGB color features, and texture features. Through BP neural network, extreme learning machine (ELM), and support vector machine (SVM) models, the importance of color, texture, and fusion features for the classification of the main roots of *Panax notoginseng* is verified. Among the three models, the SVM model performs the best, achieving an accuracy of 92.037% on the prediction set. Next, iterative retaining information variables (IRIVs), variable iterative space shrinkage approach (VISSA), and stepwise regression analysis (SRA) are used to reduce the dimension of all the features. Finally, a traditional machine learning SVM model based on feature selection and a deep learning model based on semantic segmentation are established. With the model size of only 125 kb and the training time of 3.4 s, the IRIV-SVM model achieves an accuracy of 95.370% on the test set, so IRIV-SVM is selected as the main root classification model for *Panax notoginseng*. After being optimized by the gray wolf optimizer, the IRIV-GWO-SVM model achieves the highest classification accuracy of 98.704% on the test set. The study results of this paper provide a basis for developing online classification methods of *Panax notoginseng* with different grades in actual production.

## 1. Introduction

*Panax notoginseng* is one of the most representative traditional Chinese medicines in Yunnan Province, China, and it is also known as “Jinbuhuan”. It not only has medicinal values (e.g., promoting blood circulation, removing blood stasis, relieving swelling and pain, and anti-cancer) but also has health care effects (e.g., enhancing learning and memory, improving immunity, and delaying aging) [1]. The number of heads of *Panax notoginseng* refers to the number of *Panax notoginseng* taproots per 500 g, which is the main basis for the classification of *Panax notoginseng* in the market. The weight of a single *Panax notoginseng* is the main factor that determines its selling price [2]. Currently, there are two main classification methods of *Panax notoginseng* taproots: manual classification and mechanical sorting. Manual classification is characterized by high labor intensity and low automation and efficiency, and it is often affected by subjective factors, causing misclassification and omission in the process. The mechanical sorting adopts the method of weighing, and the classification stability is low. Therefore, rapid and accurate classification of *Panax notoginseng* taproots is crucial to improve the commodity value of *Panax notoginseng*.

At present, machine vision technology has been widely used in medicine [3], industry [4], agriculture [5,6,7], aviation [8], and other fields. With the improvement of computing power and the reduction of hardware cost, a large number of studies have been conducted on the quality inspection and classification of agricultural products using machine vision technology. As for foreign studies, Huang [9] obtained the geometric features, color features, and defect areas of betel nuts. Then, betel nuts were classified by using neural network and image processing technology, with an accuracy of 90.9%. Ebrahimi et al. [10] used the ratio of long diameter to short diameter as regional indicators and hue, saturation, and intensity as color indicators to build an SVM model. The model can classify parasites and detect thrips with an error of less than 2.5%. Murat et al. [11] developed a computer vision system to obtain 16 features of bean images. In addition, they established multilayer perceptron (MLP), support vector machine (SVM), K-nearest neighbors (KNN), and decision tree (DT) classification models, and these models were compared by10-fold cross-validation and performance indicators. The overall classification accuracy of MLP, SVM, KNN, and DT were 91.73%, 93.13%, 87.92%, and 92.52%. Goncalves et al. [12] trained six convolutional neural network (CNN) architectures to detect coffee leaf miner disease and two fungal diseases. Among the architectures, Unet and DeepLabv3+ (Xception) performed the best. Wu et al. [13] used ResNet-50, ResNet 101, Xception-65, and Xception-71 to sort hydroponic lettuce. The results showed that ResNet-101 was the best, with a pixel accuracy of 99.24% and MIoU of 0.8326. In terms of speed, ResNet-50’s segmentation speed was very fast, achieving 154.0 milliseconds per image. As for domestic studies, Zhou et al. [14] designed a machine vision classification system based on a v-shaped plane mirror for online detection and classification of potato size, shape, and defects, with a classification accuracy of 91.0%. Wang et al. [15] obtained the size of Agaricus bisporus through image processing and developed a system for classification, with an accuracy of 97.42%. Dang et al. [16] established a model for the rapid and accurate detection and evaluation of potato disease degree according to the color, texture, and shape characteristic parameters of late blight spots on potato leaves. Yao et al. [17] used the improved RetinaNet model to detect rice canopy pests with an average accuracy of 93.76%. The deep multi-branch model fusion network proposed by Xie et al. [18] can identify and segment carrot defects. The experimental results indicated that the network constructed following the U-net structure achieves the best segmentation effect, with an average pixel accuracy of 96.05%. Quantitative evaluation and surface defect repair are of great significance. Yu et al. [19] used computer vision technology to obtain the image of notoginseng taproots. Then, its features such as length, width, and projected area were extracted, and a prediction model for the mass of conical notoginseng taproot and nodular notoginseng taproot was established and verified by ten-cross verification. The mean error of taproot mass of conical and nodular *Panax notoginseng* was 0.3348 and 0.4949 g, respectively. The above research results show that machine vision technology is feasible in the classification of agricultural products. However, as for grading *Panax notoginseng* taproots through machine vision technology, the amount of data is relatively small, and the feature extraction is relatively simple. In terms of the irregularity of the shape of *Panax notoginseng* taproots, accurate grading of *Panax notoginseng* requires a comprehensive consideration of various factors, such as the size, shape, texture, and color of *Panax notoginseng* taproots.

After selecting the characteristic indexes of *Panax notoginseng* taproots, this paper constructed a classification model of *Panax notoginseng* roots based on machine vision feature fusion by using traditional machine learning and deep learning techniques. Then, the prediction results of several evaluation models were compared. This method proposed in this study can classify *Panax notoginseng* of different grades accurately and rapidly, and it provides a reference for applying machine vision technology to the classification of *Panax notoginseng* taproots.

## 2. Materials and Methods

### 2.1. Test Materials

**Figure 1:**
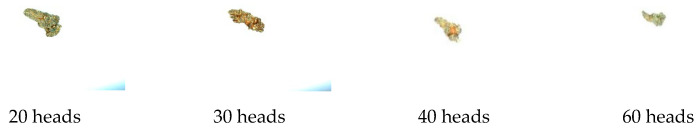
Schematic diagram of four different grades of *Panax notoginseng*.

This study takes *Panax notoginseng* planted in Wenshan City, Yunnan Province, as the research object. According to the market research, it is known that more than 90% of *Panax notoginseng* sold in the market have between 20 and 60 heads. Therefore, after preliminary treatment, 450 three-year-old “spring” *Panax notoginseng* samples with 20 heads (grade I), 30 heads (grade II), 40 heads (grade III), and 60 heads (grade IV) were selected, and the moisture content of these samples was less than 13%. The samples of different grades were weighted one by one before image information collection. The four different grades of *Panax notoginseng* main roots are shown in Figure 1.

### 2.2. The Grading Standard of Panax notoginseng Taproots

According to the national standard of Wenshan *Panax notoginseng* [20] (gbt19086-2008), the grading specifications of the four grades of the main root of notoginseng are shown in Table 1.

### 2.3. Image Acquisition System Introduction

**Figure 2:**
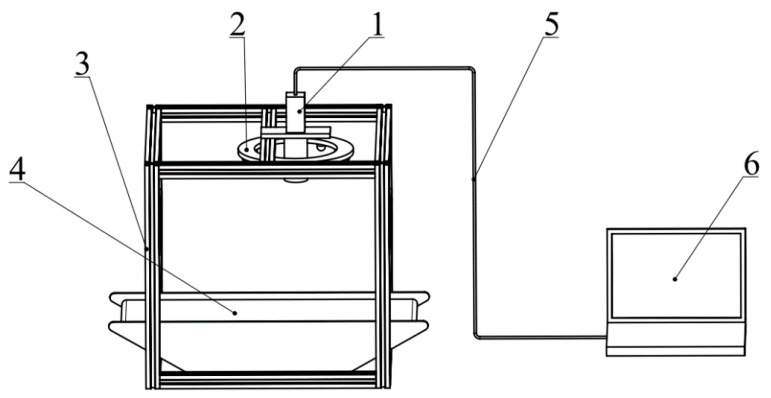
Schematic diagram of *Panax notoginseng* taproot image acquisition system. 1. Industrial camera, 2. light source, 3. camera bellows, 4. stage, 5. communication interface, 6. computer.

As shown in Figure 2, the image acquisition system consisted of a computer (Lenovo-T480), a Hikvision industrial camera (model: MV-CA013-20GC, lens: MVL-HF0828M-6MP, with a resolution of 1280 × 1024), a test chamber (400 mm × 400 mm × 400 mm), a stage, a ring-shaped LED shadowless lamp, a USB cable, and other components. The camera was fixed at the center of the top of the test box; the annular LED shadowless lamp was used as the light source, and the white high contrast was used as the background of the overhead camera; the object distance was adjusted to 27 cm, and the focal length was set to 2.8 mm. Then, the *Panax notoginseng* taproots of different grades were placed one by one on the central position of the object platform, and the shooting mode was started. The collected image data were transmitted to the computer through a USB cable, and the collected image was preprocessed using the Python language and the OpenCV machine learning framework.

### 2.4. Image Preprocessing

**Figure 3:**
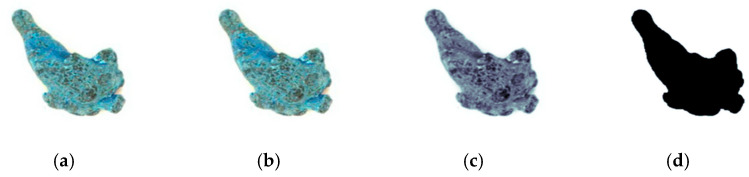
Image preprocessing. (**a**) Original image, (**b**) Gaussian filtered, (**c**) grayscale image, (**d**) binarized image.

To facilitate feature extraction and improve the algorithm efficiency, in this study, the resolution of the original image (Figure 3a) was adjusted from 1280 × 1024 to 512 × 512. Then, the image was denoised with a Gaussian filter (Figure 3b), and the filtered image was converted to grayscale (Figure 3c). Next, an open (corrode) operation with a 5 × 5 kernel function and a closed (dilate) operation were used to remove image noise, and the holes in the target were filled. Finally, the grayscale image was binarized using the Great Law (Otsu) threshold value (Figure 3d).

### 2.5. Feature Extraction

The *Panax notoginseng* taproots of different grades have obvious differences in shape, size, surface color, and texture [21,22]. Therefore, the shape, size, color, chromaticity, brightness, and saturation were selected for characterization.

#### 2.5.1. Shape and Size Feature Extraction

**Figure 4:**
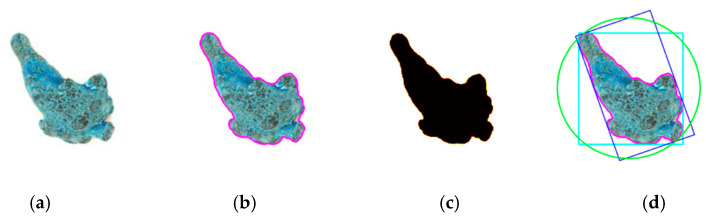
Shape feature extraction. (**a**) Gaussian filtered image, (**b**) perimeter extraction image, (**c**) area extraction image, (**d**) hybrid feature extraction image.

The shape and size features [23,24] are the basic features of the image. These features will not be affected by scaling, rotation, and translation, and they have been widely used in computer vision recognition systems. Based on the OpenCV library of Python, nine two-dimensional plane features of the *Panax notoginseng* taproots were extracted, including the mapping perimeter (the pink line in Figure 4b), the mapping area (Figure 4c), the width and height of the circumscribed rectangle (the green line in Figure 4d), and the smallest circumscribed rectangle (the width and height of the blue line in Figure 4d), the slender length (the height/width of the smallest circumscribed rectangle), the duty cycle (the area of the contour area divided by the area of the smallest circumscribed rectangle), and the radius of the smallest circumscribed circle (the green line in Figure 4d). These shape and size features were stored in the feature vector and provided to the classification model.

#### 2.5.2. Color Feature Extraction

**Figure 5:**
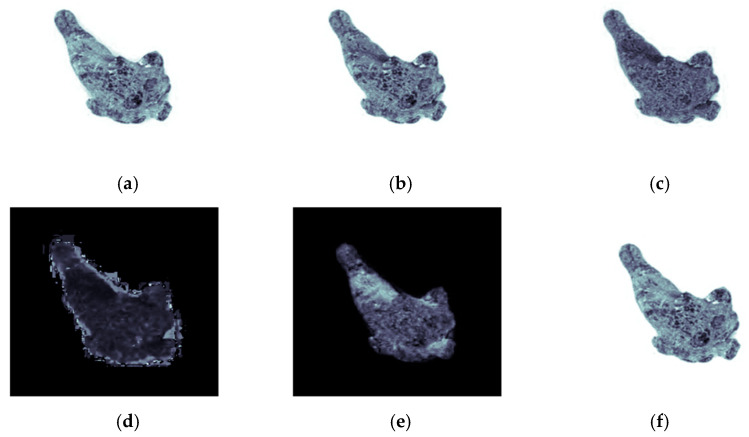
RGB and HSV visualization. (**a**) Red channel (R), (**b**) green channel (G), (**c**) blue channel (B), (**d**) hue (H), (**e**) saturation (S), (**f**) value (V).

In this study, the color moment method was adopted to extract the color features of the *Panax notoginseng* samples through two color-space models, RGB and HSV [25,26,27]. Because the color information is mainly distributed in low-order moments, the color distribution of *Panax notoginseng* taproot images was expressed by first-order moments (mean), second-order moments (variance), third-order moments (skewness), and fourth-order moments (kurtosis). Therefore, a total of 24 color features of R (Figure 5a), G (Figure 5b), B (Figure 5c) and H (Figure 5d), S (Figure 5e), and V (Figure 5f) were obtained. A visualization of a *Panax notoginseng* sample is shown in Figure 5.

The calculation formulas of RGB and HSV color characteristic parameters are as follows:

(1) $$mean=\mu_{i}=\frac{1}{N}\overset{N}{\underset{j-1}{\sum }}P_{ij}$$

(2) $$variance={\left(\frac{1}{N}\overset{N}{\underset{j=1}{\sum }}{\left(P_{ij}-\mu_{i}\right)}^{2}\right)}^{\frac{1}{2}}$$

(3) $$skewness={\left(\frac{1}{N}\overset{N}{\underset{j=1}{\sum }}{\left(P_{ij}-\mu_{i}\right)}^{3}\right)}^{\frac{1}{3}}$$

(4) $$kurtosis={\left(\frac{1}{N}\overset{N}{\underset{j=1}{\sum }}{\left(P_{ij}-\mu_{i}\right)}^{4}\right)}^{\frac{1}{4}}-3$$

In the formulas, *i* represents the three color channels R, G, and B; *j* represents the pixel value; *N* represents the number of pixels in the image; *P_ij_* represents the *i*-th color component of the *j*-th pixel of the color image, and *μ_i_* represents the average value of each color channel.

#### 2.5.3. Texture Feature Extraction

**Figure 6:**
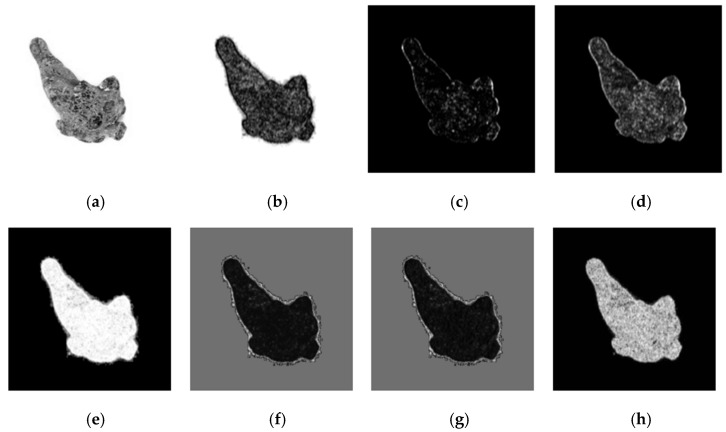
A visualization of the seven texture features. (**a**) Original image, (**b**) homogeneity, (**c**) contrast, (**d**) dissimilarity, (**e**) entropy, (**f**) energy, (**g**) correlation, (**h**) auto-correlation.

The texture feature of the image [28,29,30] reflects the external structure and organization characteristics of periodic changes on the object surface. This kind of feature is common but difficult to describe. The gray-level co-occurrence matrix (GLCM) reflects the comprehensive gray-level information about the direction, adjacent interval, and change amplitude of an image. It is widely used to convert gray values into texture information. Based on the GLCM, this study extracted seven texture features to quantitatively describe the *Panax notoginseng* taproots, including homogeneity, contrast, dissimilarity, entropy, energy, correlation, and auto-correlation. A visualization of the above seven texture features is shown in Figure 6.

The calculation formulas for the seven texture features are as follows:

(5) $$Homogeneity=\overset{M-1}{\underset{i=0}{\sum }}\overset{N-1}{\underset{j=0}{\sum }}\frac{f\left(i,j,d,\theta \right)}{1+{\left(i-j\right)}^{2}}$$

(6) $$Contrast=\overset{M-1}{\underset{i=0}{\sum }}\overset{N-1}{\underset{j=0}{\sum }}{\left(i-j\right)}^{2}f\left(i,j,d,\theta \right)$$

(7) $$Dissimilarity=\overset{M-1}{\underset{i=0}{\sum }}\overset{N-1}{\underset{j=0}{\sum }}\left|i-j\right|f\left(i,j,d,\theta \right)$$

(8) $$Entropy=-\overset{M-1}{\underset{i=0}{\sum }}\overset{N-1}{\underset{j=0}{\sum }}f\left(i,j\right){log}_{10}f\left(i,j,d,\theta \right)$$

(9) $$Energy=\overset{M-1}{\underset{i=0}{\sum }}\overset{N-1}{\underset{j=0}{\sum }}f{\left(i,j,d,\theta \right)}^{2}$$

(10) $$Correlation=\overset{M-1}{\underset{i=0}{\sum }}\overset{N-1}{\underset{j=0}{\sum }}\frac{\left(i-\mu \right)\left(j-\mu \right)f{\left(i,j,d,\theta \right)}^{2}}{\delta^{2}}$$

(11) $$Auto\_Correlation=\overset{M-1}{\underset{i=0}{\sum }}\overset{N-1}{\underset{j=0}{\sum }}\left(ij\right)f\left(i,j,d,\theta \right)$$

In these formulas, *d* represents the relative distance between the gray levels of two pixels, and it was set to 2; *θ* represents the four directions of the GLCM, i.e., 0°, 45°, 90°, and 135°. For the four directions, the average of the eigenvalues was taken as the final eigenvalue of the co-occurrence matrix. *i*, *j* = 0, 1, 2, …, *L* − 1; *L* ∈ (*M*, *N*) represents the gray level, and it was set to 64; *M* and *N* represent the size of the GLCM; *μ* represents the square root of the mean square sum in the *x*-axis and *y*-axis direction; the size of the sliding window was set to 7 × 7. Additionally, all images in the *θ* direction were separated by δ.

### 2.6. Data Processing and Model Evaluation

#### 2.6.1. Pretreatment and Dimensionality Reduction of the *Panax notoginseng* Taproot Features

After feature extraction, a total of 40 features and 1800 pieces of feature data were obtained for the 450 *Panax notoginseng* samples. However, the use of all features of *Panax notoginseng* taproots will cause data redundancy, reduce the robustness of the model, and increase the calculation amount. Feature selection can reduce the calculation amount by reducing the data dimension without affecting the prediction accuracy. In this study, three characteristic variables were adopted for feature selection, including iterative and Retaining informative variables (IRIVs), variable iterative space shrinkage approach (VISSA), and stepwise regression analysis (SRA).

IRIV [31] can statistically divide all variables into four categories: strongly sensitive variables, weakly sensitive variables, insensitive variables, and interference variables. Then, it retains strongly sensitive variables and weakly sensitive variables until there are no insensitive variables and interference variables. Based on model cluster analysis (MPA), VISSA [32] gradually optimizes spatial variables in each iteration and finally selects the optimal variable combination. The algorithm judges the error between the real value and the predicted value through interactive verification of root mean square error (RMSECV). SRA [33] eliminates insignificant variables by introducing variables into the characteristic equation one by one until all variables involved in the regression equation are significant.

#### 2.6.2. Modeling Method and Model Evaluation

The models based on traditional machine vision classification, including established BP (backpropagation) neural network [34], extreme learning machine (ELM) [35], and support vector machine (SVM) [36], and the deep learning models, including the segmented U-net [37], PSPNet [38], and DeepLabv3+ [39], are used as the main root classification models for different head numbers.

SVM is a supervised classification method with good generalization ability. It can effectively deal with linear and nonlinear data and can be used for regression, classification, etc. In this paper, radial basis function (RBF) was used as the kernel function of SVM. The parameters were optimized using genetic algorithm (GA), particle swarm optimization (PSO), and gray wolf optimizer (GWO). The three intelligent optimization algorithms all use five-fold cross-validation.

PSPNet is a classic semantic segmentation network, and it can effectively extract and classify image data features. The core module is the pyramid pooling module, which can aggregate the context information of different regions, thereby improving the acquisition of global information. In this article, PSPNet automatically extracted and classified the characteristics of the main roots of *Panax notoginseng*. The quality of the model was evaluated by average pixel accuracy (MPA) and average intersection and ratio (MIoU).

MATLAB 2019a, PyCharm, and OpenCV software were used for sampling, feature extraction, feature selection, model establishment, and analysis of the original data. Prediction accuracy was taken as the performance evaluation metric of the models. The higher the value of prediction accuracy, the better the performance of the model.

## 3. Test Results and Analysis

### 3.1. Comparison of Fusion Feature Classification Models in Different Dimensions

In this paper, there were 1800 samples in total, and the ratio of the training set to test set was 7:3. Thus, 1260 training set samples (315 *Panax notoginseng* taproots at different grades) were randomly selected, and the remaining 540 samples were used as test set (135 *Panax notoginseng* taproots of different grades). Through the three network models of BP, ELM, and SVM, shape and size (9 features); shape, size, and texture (16 features); shape, size, and color (33 features); and shape, size, texture, and color (40 types of features) were respectively analyzed. The fusion features of four different situations and different dimensions were compared and analyzed, and the results are shown in Table 2.

The following observations can be made from Table 2:

(1) When shape and size were taken as the features, among the three classification models, SVM achieved the highest accuracy, with an accuracy of 76.467% on the training set and an accuracy of 75.185% on the test set.
(2) When shape, size, and texture were taken as the features, on the test set, the accuracy of the BP neural network was 14.782% higher than that without texture features; the accuracy of ELM was 17.222% higher than that without texture features; the accuracy of SVM was 15.222% higher than that without texture features. Thus, texture features were important in the main root classification of *Panax notoginseng*. Among the three classification models, SVM achieved the highest accuracy on the training set and test set.
(3) When shape, size, and color were taken as the features, on the test set, the accuracy of the BP neural network was 23.309% higher than that without color features; the accuracy of ELM was 36.297% higher than that without texture features; the accuracy of SVM was 16.111% higher than that without texture features. Thus, color features were important in the classification of the main root of *Panax notoginseng*. Among the three classification models, SVM achieved the highest accuracy on the training set and test set.
(4) When shape, size, texture, and color were taken as the features, on the test set, the accuracy of the BP neural network was 1.47% higher than that with color features without texture features, and it was 10.005% higher than that with texture features without color features; the accuracy of ELM was 5.135% higher than that with color features without texture features, and it was 24.21% higher than that with texture features without color features; the accuracy of SVM was 0.741% higher than that with color features without texture features, and it was 1.63% is higher than that with texture features without color features.

The above results show that the *Panax notoginseng* main root classification model established on fusion features was the best, and the accuracy of the SVM model on the training set and test set was, respectively, 91.191% and 92.037%, both of which were the highest. Therefore, SVM was selected as the classification model of the main root of *Panax notoginseng*.

### 3.2. Comparison and Analysis of Different Feature Selection Methods

If there are too many features in the process of model building, the robustness of the model will be reduced. Moreover, too much redundant information will be introduced, which will prolong the time of data processing. If there are too few features, it will cause information loss and reduce the prediction accuracy of the model. Therefore, the extracted feature data were optimized first, and the best feature combination was selected for model building.

#### 3.2.1. Selection of Characteristic Variables Based on IRIV

**Figure 7:**
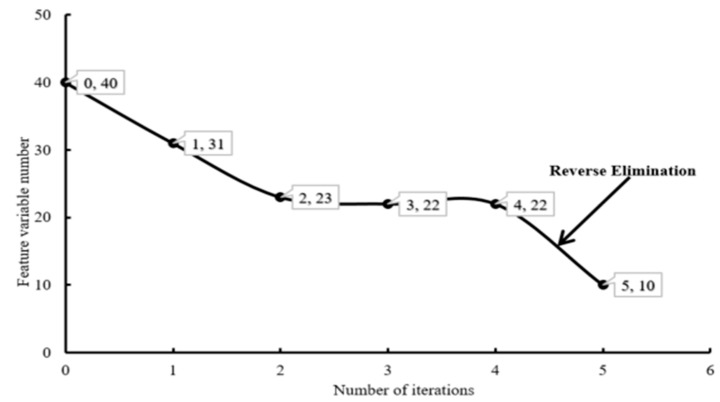
IRIV variable selection results based on image shape, size, color, and texture information.

This study exploited the IRIV algorithm to reduce the dimensions of the shape, size, color, and texture features of the taproots of different grades. The result is shown in Figure 7. After four iterations, the feature variables were reduced from 40 to 22. Then, the elimination strategy was performed on the remaining features to calculate the RMSECV. When the smallest RMSECV was achieved, the remaining features were eliminated from 22 to 12. Finally, ten feature variables were determined, which were projected area (A), slender length (E), R skewness (Rs), R kurtosis (Rk), B average (Bmean), S average (Smean), S variance (Svar), S skewness (Ss), contrast, and auto-correlation.

The calculation formula of *RMSECV* is as follows:

(12) $$RMSECV=sqrt\left(sum\left(\frac{{\left(Y-Y_{v}\right)}^{2}}{n}\right)\right)$$

where *Y* is the real value; *Y_V_* is the predicted value during cross-validation, and *n* is the number of samples.

#### 3.2.2. Selection of Characteristic Variables Based on VISSA

**Figure 8:**
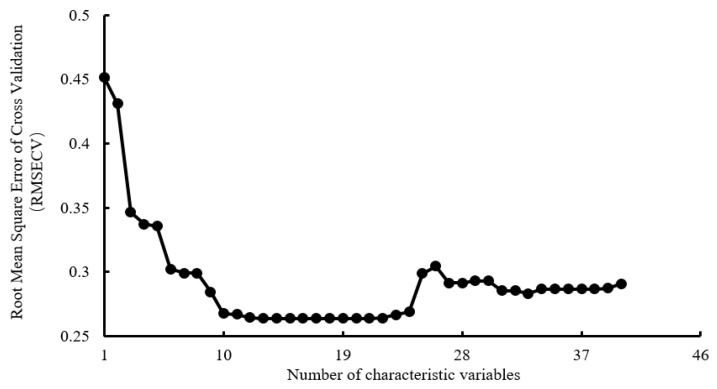
VISSA variable selection results based on image shape, size, color, and texture information.

In VISSA feature selection, the variable subset generated by weighted binary sampling (WBMS) was set to 500. In this case, the initial sampling weight was 0.5, and the proportion of the subset model was 5%, leading to the best feature combination. It can be seen from Figure 8 that when the number of characteristic variables was less than 13, the *RMSECV* gradually decreased as the number of selected variables increased. When the number of feature variables was between 13 and 21, *RMSECV* remained at 0.2639. When the number of features was greater than 21, *RMSECV* increased. Therefore, 13 to 21 features could be selected. Through VISSA feature selection, it was found that the following 21 feature combinations were optimal, i.e., the projected area (A), the width of the smallest bounding rectangle (Min_w), the slender length (E), the skewness of R (Rs), and the variance of G (Gvar), the variance of B (Bvar), the skewness of B (Bs), the variance of H (Hvar), the skewness of H (Hs), the average of S (Smean), the variance of S (Svar), S skewness (Ss), S kurtosis value (Sk), V mean value (Vmean), V skewness (Vs), V kurtosis value (Vk), energy, contrast, homogeneity, dissimilarity, and auto-correlation.

#### 3.2.3. Selection of Characteristic Variables Based on SRA

**Figure 9:**
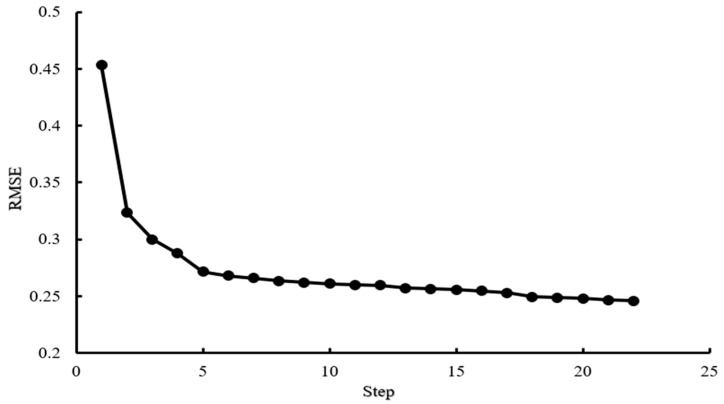
Iterative root mean square error change trend graph.

Based on the independent variable’s influence on the dependent variable, SRA introduces significant independent variables into the regression equation and eliminates insignificant independent variables from the regression equation one by one until no variables are introduced or eliminated. As shown in Figure 9, when there were 22 characteristic variables, RMSE was the minimum. This indicates that the fitting effect between the observed values and real values was good and that the 22 features had a great influence on classifying *Panax notoginseng* taproots. Therefore, these 22 features were extracted through the SRA algorithm as the optimal combination of feature variables, including projected area (A), the slender length (E), the radius of the smallest circumscribed circle (Radius), the average value of R (Rmean), R variance (Rvar), G average (Gmean), G variance (Gvar), G skewness (Gs), B average (Bmean), H average (Hmean), H variance (Hvar), the skewness of H (Hs), the kurtosis value of H (Hk), the variance of S (Svar), the kurtosis value of S (Sk), the average value of V (Vmean), the value of V Variance (Vvar), V skewness (Vs), V kurtosis (Vk), entropy, contrast, and auto-correlation.

### 3.3. Establishment of the Classification Model

#### 3.3.1. Establishment of SVM Classification Model Based on Feature Selection

IRIV, VISSA, and SRA were, respectively, adopted for feature selection to establish an SVM classification model for *Panax notoginseng* taproots. The established models were compared and analyzed, and the results are listed in Table 3.

It can be seen from Table 3 that after feature selection, the number of feature variables was reduced, and the accuracy of the model on the training set and test set was higher than that of the model established with full features. Therefore, feature selection for full features was essential. In addition, the IRIV-SVM model achieved an accuracy of 95.370% on the test set, which was higher than that of VISSA-SVM and SRA-SVM. It shows that the combined feature variables extracted by IRIV could better reflect the characteristics of *Panax notoginseng* taproots of different grades. Therefore, this paper choose IRIV-SVM as the classification model for *Panax notoginseng* taproots.

#### 3.3.2. The Establishment of Deep Learning Network Model Based on Semantic Segmentation

In recent years, deep learning methods have performed well in the classification, detection, and segmentation of agricultural products. Compared with traditional machine learning technology, deep learning networks can automatically extract image features layer by layer from images and then classify and recognize the features through classifiers, which avoids the data preparation and manual feature selection of traditional machine learning algorithms. Furthermore, the extracted image features are richer, and the model performance is better.

**Figure 10:**
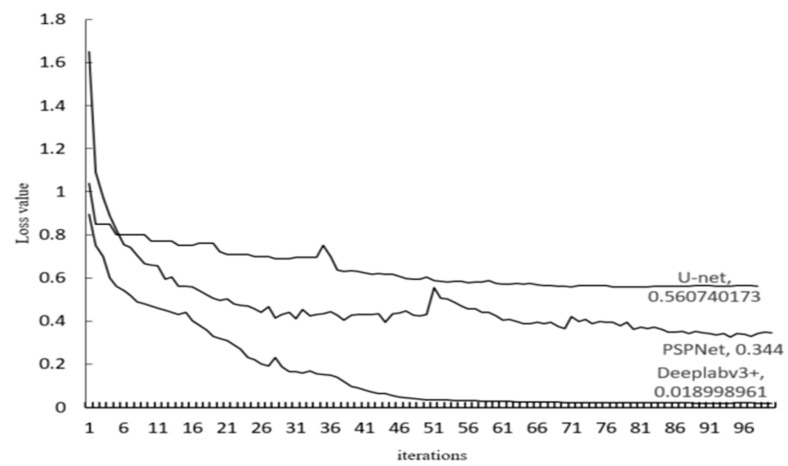
Loss value curve changes with iterations.

In this paper, three semantic segmentation networks, PSPnet, U-net, and DeepLabv3+ were selected as the classification models for *Panax notoginseng* main root with different head counts. Meanwhile, three types of convolutional neural networks, including ResNet50, VGG16, and MobileNetV2, were used as feature extraction networks. Among them, VGG16 consists of 13 convolutional layers and 3 fully connected layers, a total of 16 floors. ResNet50 introduces the residual network, and there are two basic blocks, i.e., Identity Block and Conv Block. Identity Block uses 1 × 1 convolution to reduce the dimensionality before the 3 × 3 network structure and uses 1 × 1 convolution to increase the dimensionality after the 3 × 3 network structure. Compared with the direct use of 3 × 3 network convolution, Identity Block achieves a better effect with fewer parameters. Moreover, the input and output have the same dimension, and they can be connected in series to deepen the network. The input and output of Conv Block have different dimensions and they cannot be directly connected in series. The function of Conv Block is to change the dimension of the network. The MobileNetV2 network uses an inverted residual structure. It uses 1 × 1 convolution to increase the dimension before the 3 × 3 network structure and uses 1 × 1 convolution to reduce the dimension after the 3 × 3 network structure. All model training and testing were performed under the same hardware and software environment. The hardware platform was equipped with Intel(R) Xeon(R) Gold 5218 CPU, 64GB RAM, 512GB SK hynix SC401 SATA disk, and NVIDIA RTX 2070 GPU. The software included Tensorflow-GPU 1.13.2 deep learning framework, Python 3.6 language, and Windows operating system. There were 1800 samples in total, and the ratio of the training set to test set was determined to be 8:2 by experimentation. The loss curve of the three models is shown in Figure 10.

As can be seen from Figure 10, the U-net model was quickly fitted in the first five iterations, and the loss value decreased rapidly. During 5–61 iterations, the loss value decreased slowly, and the variation was extremely large during 34–36 iterations. After 61 iterations, the loss value gradually stabilized at about 0.56. The PSPNet model was quickly fitted in the first 31 iterations, and the loss value decreased rapidly. During 31–80 iterations, the loss value decreased slowly and the variation was extremely large during 50–60 iterations. After 80 iterations, the loss value gradually stabilized at about 0.344. Before the 50th iteration, the loss value of the DeepLabv3+ model gradually decreased, and the model gradually converged. After the 50th iteration, the loss value gradually stabilized at about 0.019.

In the training process, a total of 100 weights were output in each iteration. It is not that the model performance increases with the number of iterations, but too much training may lead to overfitting, so the trained model needs to be tested and evaluated. Taking category pixel average accuracy rate (MPA) and average interaction ratio (MIoU) as the metrics, the evaluation results are listed in Table 4.

It can be seen from Table 4 that PSPNet achieved the best overall performance, and the MPA and MIoU were, respectively, 77.98% and 88.97% on the test set. Although PSPNet did not have the decoder module of the DeepLabv3+ model feature extraction network, and the number of feature extraction network layers was relatively small, it achieved 2.73% higher accuracy than DeepLabv3+ with fewer feature extraction network layers and parameters. This result indicates that too many network layers will indeed extract a lot of features, but the network model is large, and the training time is long; also, there are too many features that are useless for classifying the main root of *Panax notoginseng*. This leads to a decrease in the accuracy of the network. The MIoU of U-net was only 80.98%, which was lower than that of PSPNet and DeepLabv3+. This was because U-net had fewer feature extraction network layers. Though DeepLabv3+ had a large model, its performance was moderate in terms of MPA and MIoU. To sum up, as a segmentation network for the main roots of different grades, the ResNet50 convolutional neural network in the PSPNet model achieved better performance and was suitable for grading the main roots of notoginseng.

### 3.4. Model Comparison

According to Section 3.3.1, among the traditional machine learning models based on feature selection, the IRIV-SVM model achieved an accuracy of 94.048 on the training set and an accuracy of 95.370% on the test set, both of which were the highest. According to Section 3.3.2, among the deep learning models based on semantic segmentation, PSPNet achieved an MPA of 77.98% and an MIoU of 88.97%, both of which were the highest.

Compared with IRIV-SVM, PSPNet automatically extracts features from the image layer by layer, so it can avoid the problem of manual feature selection. However, the model size of PsPNet was 0.65 G, and the training time was 9 h, which has a high requirement on the hardware platform. Though the IRIV-SVM algorithm involves data preparation and manual feature selection, its model size was only 125 kb, the training time was 3.4 s, and the accuracy was high. Therefore, IRIV-SVM was chosen as the classification model for *Panax notoginseng* taproot.

### 3.5. Optimization of IRIV-SVM Hierarchical Model

Since the classification accuracy of SVM is determined by c (penalty factor) and g (kernel function parameter) [40], the default setting of c and g may not lead to the best performance. Therefore, the interference to the model performance can be eliminated by adjusting the values of c and g. In this paper, the IRIV-SVM model was optimized by five-fold cross-validation and three intelligent optimization algorithms, i.e., GWO, GA, and PSO. After experimentation, the maximum number of iterations of the three optimization algorithms was set to 100, and the population size was set to 20. In GWO, the search range of c and g were both set to [0.01, 100]. In GA, the search range of c was set to [0, 100], and the search range of g was set to [0, 1000]. Furthermore, the crossover probability was set to 0.6, and the mutation probability was set to 0.03. In PSO, the search range of c was set to [0.1, 100], and the search range of g was set to [0.01, 1000].

**Figure 11:**
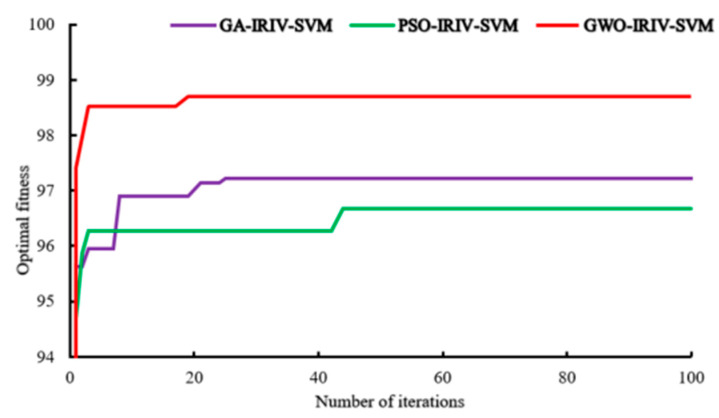
Comparison of fitness trends of three intelligent optimization algorithm construction models.

It can be seen from Figure 11 that after 100 iterations, the three optimization algorithms all achieved the best fitness. The GWO algorithm converged to the best fitness of 98.704% in the 19th iteration; the GA algorithm converged to the best fitness of 97.220% in the 25th iteration, and the PSO algorithm converged to the best fitness of 96.67% in the 44th iteration. Comparative analysis indicates that the GWO algorithm had the fastest convergence speed and the best adaptability.

It can be seen from Table 5 that after optimization by the three optimization algorithms of GWO, GA, and PSO, the accuracy of the IRIV-SVM model on the test set increased by 3.334%, 3.149%, and 2.408%, respectively. Comparative analysis indicates that IRIV-GWO-SVM achieved an accuracy of 98.703% on the test set, which was higher than that of IRIV-GA-SVM and IRIV-PSO-SVM. Therefore, this study chose the IRIV-GWO-SVM model for *Panax notoginseng* taproot classification.

### 3.6. Model Validation

**Figure 12:**
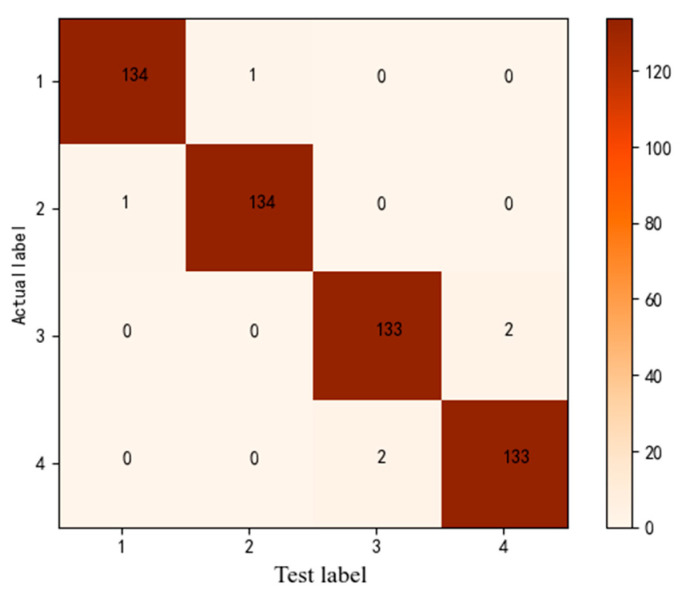
The confusion matrix of the *Panax notoginseng* taproots of different grades.

The confusion matrix of IRIV-GWO-SVM for classifying the *Panax notoginseng* taproots at different grades is shown in Figure 12. The accuracy of IRIV-GWO-SVM for classifying grade-I, grade-II, grade-III, and grade-IV *Panax notoginseng* taproots was, respectively, 99.259%, 99.259%, 98.510%, and 98.510%, with an average classification accuracy of 98.8845%. In summary, the IRIV-GWO-SVM model achieved good classification accuracy for *Panax notoginseng* taproots of different grades.

## 4. Discussion

In this study, 450 *Panax notoginseng* taproots at four different grades were selected. The use of sufficient and diverse samples makes the model more convincing. Considering the irregularity of the shape of *Panax notoginseng* taproot, the characteristics of size, shape, texture, color, and other characteristics that affect the classification of *Panax notoginseng* taproot were extracted. This solves the problem that the previously extracted features are relatively simple and cannot accurately express the characteristics of *Panax notoginseng* taproots. Deep learning can automatically extract image features layer by layer from images through convolutional neural networks and then perform classification and recognition through classifiers. This research established a deep learning classification model based on PSPnet, U-net, and DeepLabv3+ for semantic segmentation of different heads and notoginseng roots. Meanwhile, three types of convolutional neural networks including ResNet50, VGG16, and MobileNet were used as feature extraction networks. Taking the category average pixel accuracy and average interaction ratio as evaluation indicators, the traditional machine learning and deep learning grading models were compared and analyzed, and the best model was selected and optimized. The accuracy of the optimized model reached 98.704% on the test set.

## 5. Conclusions

In this study, the IRIV-GWO-SVM classification model achieved rapid and non-destructive classification of *Panax notoginseng* taproots, which provides an effective method for the classification of irregular agricultural products.

(1) A model based on image feature fusion was established for classifying *Panax notoginseng* taproots. By preprocessing the taproot images of *Panax notoginseng* collected using a CCD camera, 40 features such as shape, size, color, and texture were extracted. Through the classification accuracy of the three classification models of BP, ELM, and SVM, the importance of color, texture, and fusion features to the classification model of *Panax notoginseng* root was proved. Three feature selection algorithms, i.e., IRIV, VISSA, and SRA, were used to reduce the dimension of the whole feature. After feature selection, 22, 21, and 10 optimal feature variable combinations were obtained respectively, and redundant feature data were eliminated.
(2) Deep learning can automatically extract image features layer by layer from images through convolutional neural networks and then perform classification and recognition through classifiers. This research established a deep learning classification model for *Panax notoginseng* taproots and selected PSPnet, U-net, and DeepLabv3+ for semantic segmentation. Meanwhile, three types of convolutional neural networks including ResNet50, VGG16, and MobileNet were used as feature extraction networks. Additionally, the average pixel accuracy and average interaction ratio of the categories were used as evaluation indicators. The results show that the PSPNet model achieved the best overall performance, with MPA of 77.98% and MioU of 88.97% on the test set.
(3) The traditional machine learning SVM classification model based on feature selection and the deep learning model based on semantic segmentation were established. Among the traditional machine learning SVM classification models, the IRIV-SVM model achieved an accuracy of 94.048% on the training set and an accuracy of 95.370% on the test set, both of which were the highest. Among the deep learning models, PSPNet achieved a MAP of 77.98% and a MioU of 88.97%, both of which were the highest. Compared with IRIV-SVM, PSPNet automatically extracts image features layer by layer. It avoids manual feature selection, but its model size was 0.65 G, and the training time was 9 h, which has high requirements for the hardware equipment. Though IRIV-SVM involves data preparation and manual feature selection, its model as only 125 kb, and the training time was 3.4 s. While the number of features was reduced, it effectively increased the efficiency and accuracy of the model. Therefore, IRIV-SVM was chosen as the classification model of *Panax notoginseng* taproot.
(4) The GWO, GA, and PSO algorithms were introduced to optimize the IRIV-SVM model. The results show that the IRIV-GWO-SVM classification model achieved the best optimization effect. The classification accuracy was 98.704% on the test set, and the optimization effect was increased by 3.334%.

In this study, a new classification method of *Panax notoginseng* taproot is proposed, and a classification model based on machine vision feature fusion is established for *Panax notoginseng* taproot of different grades. The results show that the proposed model is feasible, which provides a theoretical reference for the subsequent development of *Panax notoginseng* taproot classification system based on machine vision.

## Figures and Tables

**Table 1:** Grading specification of taproot of *Panax notoginseng*.

Grades	Root Weight/g	Number of Heads/pcs
Grade 1	≥25.0	≤20
Grade 2	≥17.0	≤30
Grade 3	≥12.5	≤40
Grade 4	≥8.5	≤60

**Table 2:** Comparison of classification models based on different fusion dimensions.

Model	Fusion Feature	Feature Number/pcs	Training Set Accuracy/%	Test Set Accuracy/%
BP	Shape, size	9	67.963	65.234
ELM	Shape, size	9	41.825	35.185
SVM	Shape, size	9	76.746	75.185
BP	Shape, size, texture	16	83.889	80.016
ELM	Shape, size, texture	16	53.095	52.407
SVM	Shape, size, texture	16	89.508	90.407
BP	Shape, size, color	33	89.630	88.542
ELM	Shape, size, color	33	73.254	71.482
SVM	Shape, size, color	33	90.793	91.296
BP	Shape, size, texture, color	40	90.741	90.021
ELM	Shape, size, texture, color	40	79.683	76.617
SVM	Shape, size, texture, color	40	91.191	92.037

**Table 3:** Comparison of SVM classification models based on feature selection.

Model	Model Size/kb	Training Time/s	Feature Number/pcs	Training Set Accuracy/%	Test Set Accuracy/%
IRIV-SVM	125	3.4	10	94.048	95.370
VISSA-SVM	264	0.371	21	92.222	92.778
SR-SVM	270	0.318	22	91.984	92.963

**Table 4:** Comparison of three different semantic segmentation networks.

Model	Model Size/G	Training Time/h	MPA/%	MIoU/%
U-net	0.21	5.5	69.21	80.89
PSPNet	0.65	9	77.98	88.97
DeepLabv3+	1.05	11.5	75.89	86.24

**Table 5:** Comparison of the IRIV-SVM models optimized by different intelligent optimization algorithms.

Model	Training Set Accuracy/%	Test Set Accuracy/%	Best c	Best g
IRIV-GWO-SVM	97.460	98.704	67.889	0.201
IRIV-GA-SVM	97.540	98.519	60.138	0.224
IRIV-PSO-SVM	97.937	97.778	100.000	0.836

## Data Availability

Not applicable.
